# Longitudinal assessment of optic nerve head changes using optical coherence tomography in a primate microbead model of ocular hypertension

**DOI:** 10.1038/s41598-020-71555-0

**Published:** 2020-09-07

**Authors:** Anita S. Y. Chan, Tin Aung Tun, John C. Allen, Myoe Naing Lynn, Sai Bo Bo Tun, Veluchamy Amutha Barathi, Michaël J. A. Girard, Tin Aung, Makoto Aihara

**Affiliations:** 1 Singapore Eye Research Institute and Singapore National Eye Centre, 11 Third Hospital Avenue, Singapore, 168751 Singapore; 2 Department of Ophthalmology, University of Tokyo, Tokyo, Japan; 3 Ophthalmic Engineering & Innovation Laboratory (OEIL), Singapore Eye Research Institute, Singapore, Singapore; 4 Duke-NUS Medical School, Singapore, Singapore; 5 Department of Ophthalmology, Yong Loo Lin School of Medicine, National University of Singapore, Singapore, Singapore

**Keywords:** Biological techniques, Experimental models of disease, Preclinical research

## Abstract

In humans, the longitudinal characterisation of early optic nerve head (ONH) damage in ocular hypertension (OHT) is difficult as patients with glaucoma usually have structural ONH damage at the time of diagnosis. Previous studies assessed glaucomatous ONH cupping by measuring the anterior lamina cribrosa depth (LCD) and minimal rim width (MRW) using optical coherence tomography (OCT). In this study, we induced OHT by repeated intracameral microbead injections in 16 cynomolgus primates (10 unilateral; 6 bilateral) and assessed the structural changes of the ONH longitudinally to observe early changes. Elevated intraocular pressure (IOP) in OHT eyes was maintained for 7 months and serial OCT measurements were performed during this period. The mean IOP was significantly elevated in OHT eyes when compared to baseline and compared to the control eyes. Thinner MRW and deeper LCD values from baseline were observed in OHT eyes with the greatest changes seen between month 1 and month 2 of OHT. Both the mean and maximum IOP values were significant predictors of MRW and LCD changes, although the maximum IOP was a slightly better predictor. We believe that this model could be useful to study IOP-induced early ONH structural damage which is important for understanding glaucoma pathogenesis.

## Introduction

Glaucoma is the second leading cause of blindness in the world^1^ and is characterised by the progressive loss of retinal ganglion cells (RGC), thinning of the retinal nerve fibre layer (RNFL), atrophy of the optic nerve head (ONH) and loss of vision^1,2^. In primary open angle glaucoma (POAG), chronic intraocular pressure (IOP) elevation or ocular hypertension (OHT) induces neural and connective tissue deformation and/or remodelling within the ONH that can lead to permanent structural damage^3–6^. In humans, early structural damage within the ONH at the onset of chronic IOP elevation is difficult to characterise as patients with POAG usually have existing structural damage at the time of diagnosis^1,2^. Moreover, patients with OHT progress slowly^2^. Thus, primate models of OHT allow us to assess the changes in the ONH longitudinally and determine the time course of early ONH structural damage in response to IOP^7–11^.

In primate OHT models, laser photocoagulation of the trabecular meshwork is conventionally used to generate experimental glaucoma (EG)^7,9^. However, the laser OHT model requires expensive laser equipment, laser expertise, and laser-related safety and licensing^7,9^. Another disadvantage of the laser model is the accompanying inflammation that is triggered by laser damage to the trabecular meshwork, which can hinder ONH and retinal imaging^12^. Obstruction of the trabecular meshwork by injecting microbeads was first used in rodents to create an OHT or EG model^12–14^. The microbeads or microspheres used in rodent studies were made from various materials, such as polysterene or latex, and can have fluorescent properties to facilitate visualisation^14^, or magnetic properties to aid their distribution to the angle^12^. Although useful, our interpretations made from the rodent model are limited as the retinal and ONH structures markedly differ from those found in humans. Furthermore, ONH imaging is difficult to achieve in such small eyes. Consequently, the microbead model of OHT in primates was first introduced in rhesus monkeys^10^ and more recently in squirrel monkeys^15^ as a potential model for EG. The structure and function of the retina and the ONH is highly conserved between primates and humans; as such, primate models are useful for examining drug metabolism and the pathogenesis of human eye diseases^15–17^. Moreover, the IOP in primates is similar to that observed in affected humans^18^. Additionally, in the primate microbead OHT model, inflammation of the anterior segment of the eye has not been reported^10,15,19^ and the procedure is less expensive and can be performed by both clinicians and researchers^8,10,12–15,19^.

The ONH changes induced by EG can be monitored by assessing the lamina cribrosa depth (LCD) and minimum rim width (MRW) using spectral-domain (SD) optical coherence tomography (OCT) in the laser-induced primate OHT model. The MRW (the width of the neuroretinal rim at the ONH) correlates well with the RNFL thickness^4,5,20–23^. Both thinning of the MRW and deepening of the LCD occurs earlier than RNFL thinning in the laser-induced primate OHT model; as such, these parameters have been used to detect early structural ONH changes^20,21,23^.

IOP is the only modifiable risk factor for glaucoma and is associated with glaucoma development and progression^24,25^. In addition, mounting evidence suggests that IOP exerts a mechanical load on the ONH and adjacent tissues, which can lead to ONH damage in glaucoma^3,11,26^. However, IOP itself varies considerably over a 24-h cycle and thus it is important to identify which aspect of IOP fluctuations is most important for the clinician to monitor and control to prevent glaucoma progression^2,25^. Thus, attempts to characterize these IOP fluctuations or variables have been made using parameters such as mean, maximum and standard deviation of IOPs^24,25^. In the primate laser model, the maximum IOP seems to be the best predictor of ONH structural change^24^.

In this study, we aimed to elucidate the early ONH changes induced by raised IOP in the microbead OHT primate model and the IOP characteristics influencing such changes. We believe that establishing the microbead OHT model in primates as an alternative to the laser model will be useful to study glaucoma pathogenesis and provide avenues for new therapies.

## Results

### Baseline IOP and primate demographics

All primates (n = 16) were female and aged 4–9 years. The mean IOP value for the right eye did not statistically differ from that of the left eye in each primate at baseline (12 ± 2.4 mmHg vs 12 ± 2.2 mmHg in Group 1 and 14 ± 1.6 mmHg vs 15 ± 1.7 mmHg in Group 2, both P > 0.05) (Table 1). In group 1, the right eye mean baseline IOP (12.0 ± 2.4 mmHg) was not statistically different from the group 2 right eye mean baseline IOP (14.0 ± 1.6 mmHg, p = 0.117) although a 2 mmHg difference was noted. A small but significant difference of 3 mmHg was noted between the left eye mean baseline IOP in Group 1 (12.0 ± 2.2 mmHg) when compared to the Group 2 left eye mean baseline IOP (15.0 ± 1.7 mmHg, p = 0.014).

**Table 1:** Demographics of the primates with intraocular profiles at baseline and after microbead induction of Ocular Hypertension.

ID	Sex	Age, years	Baseline IOP, mmHg (mean ± SD)		Number of injections for IOP elevation	The IOP elevation from first injection, weeks	IOP (months 2–7), mmHg (mean ± SD)		IOP (months 2–7), mmHg (maximum ± SD)	IOP (months 2–7), mmHg (maximum ± SD)
			Right eye	Left eye			Right eye	Left eye	Right eye	Left eye
**Group 1**										
3914	Female	9	16.0 ± 0.5	14.0 ± 1.0	5	4	46.0 ± 16.9	24.0 ± 1.9	59.0 ± 20.1	27.0 ± 3.1
5186	Female	9	10.0 ± 0.9	10.0 ± 1.2	7	6	40.0 ± 14.3	15.0 ± 1.5	51.0 ± 21.3	18.0 ± 1.9
5187	Female	9	9.0 ± 0.4	9.0 ± 0.4	7	6	32.0 ± 18.3	15.0 ± 1.4	44.0 ± 24.7	17.0 ± 1.9
5189	Female	9	10.0 ± 0.6	10.0 ± 0.9	8	7	47.0 ± 16.6	16.0 ± 1.4	55.0 ± 18.8	18.0 ± 1.2
5191	Female	9	9.0 ± 0.5	12.0 ± 0.5	6	5	53.0 ± 16.0	17.0 ± 1.1	61.0 ± 19.8	20.0 ± 1.6
5192	Female	5	12.0 ± 1.0	11.0 ± 1.0	6	5	38.0 ± 18.7	21.0 ± 1.2	49.0 ± 16.7	23.0 ± 1.0
5194	Female	9	13.0 ± 0.5	11.0 ± 0.5	7	6	41.0 ± 13.6	17.0 ± 1.3	49.0 ± 20.2	19.0 ± 2.3
5195	Female	9	12.0 ± 0.6	12.0 ± 1.2	6	5	43.0 ± 18.2	17.0 ± 1.2	54.0 ± 17.3	20.0 ± 2.3
5234	Female	9	15.0 ± 0.6	14.0 ± 0.6	5	4	38.0 ± 14.1	17.0 ± 1.0	53.0 ± 17.3	19.0 ± 0.6
5235	Female	9	14.0 ± 0.6	16.0 ± 0.6	6	5	50.0 ± 15.2	19.0 ± 1.4	61.0 ± 16.2	20.0 ± 0.8
Group 1			12.0 ± 2.4	12.0 ± 2.2	6 ± 1	5 ± 1	43.0 ± 6.3	18.0 ± 2.8	54.0 ± 5.6	20.0 ± 3.0
**Group 2**										
2677	Female	5	13.0 ± 1.2	15.0 ± 1.7	6	5	26.0 ± 11.5	24.0 ± 9.2	33.0 ± 8.30	38.0 ± 10.8
2705	Female	5	16.0 ± 1.7	15.0 ± 2.3	6	5	29.0 ± 10.7	23.0 ± 6.7	27.0 ± 7.2	39.0 ± 10.5
5407	Female	5	14.0 ± 1.9	12.0 ± 1.7	3	2	35.0 ± 21.0	37.0 ± 16.3	46.0 ± 21.5	50.0 ± 16.4
5408	Female	5	15.0 ± 0.5	14.0 ± 0.6	6	5	32.0 ± 10.5	32.0 ± 10.9	38.0 ± 9.3	37.0 ± 12.2
2873	Male	4	16.0 ± 2.9	17.0 ± 2.1	6	5	31.0 ± 15.6	38.0 ± 17.1	53.0 ± 16.9	41.0 ± 15.1
2874	Male	4	12.0 ± 2.3	16.0 ± 1.0	5	4	33.0 ± 13.9	32.0 ± 10.3	39.0 ± 11.5	41.0 ± 15.4
Group 2			14.0 ± 1.6	15.0 ± 1.7	5 ± 1	4 ± 1	31.0 ± 3.2	31.0 ± 6.3	39.0 ± 9.2	41.0 ± 4.7

*ID* primate identification number, *IOP* intraocular pressure, mmHg, *SD* standard deviation.

### Microbead induction of ocular hypertension

Initial IOP elevation was achieved with a mean of 6 ± 1 microbead injections in Group 1 primates and 5 ± 1 injections in Group 2 primates (Table 1). An average of 5 ± 1 weeks in Group 1 and 4 ± 1 weeks in Group 2 was needed from the first injection to reach an IOP > 20 mmHg (Table 1). The mean IOP in OHT eyes (measured once the IOP was > 20 mmHg) in both groups is reported in Table 1. After the initial 4–6 injections, no further injections were required to maintain OHT until month 7 (end-of-study).

**Figure 1:**
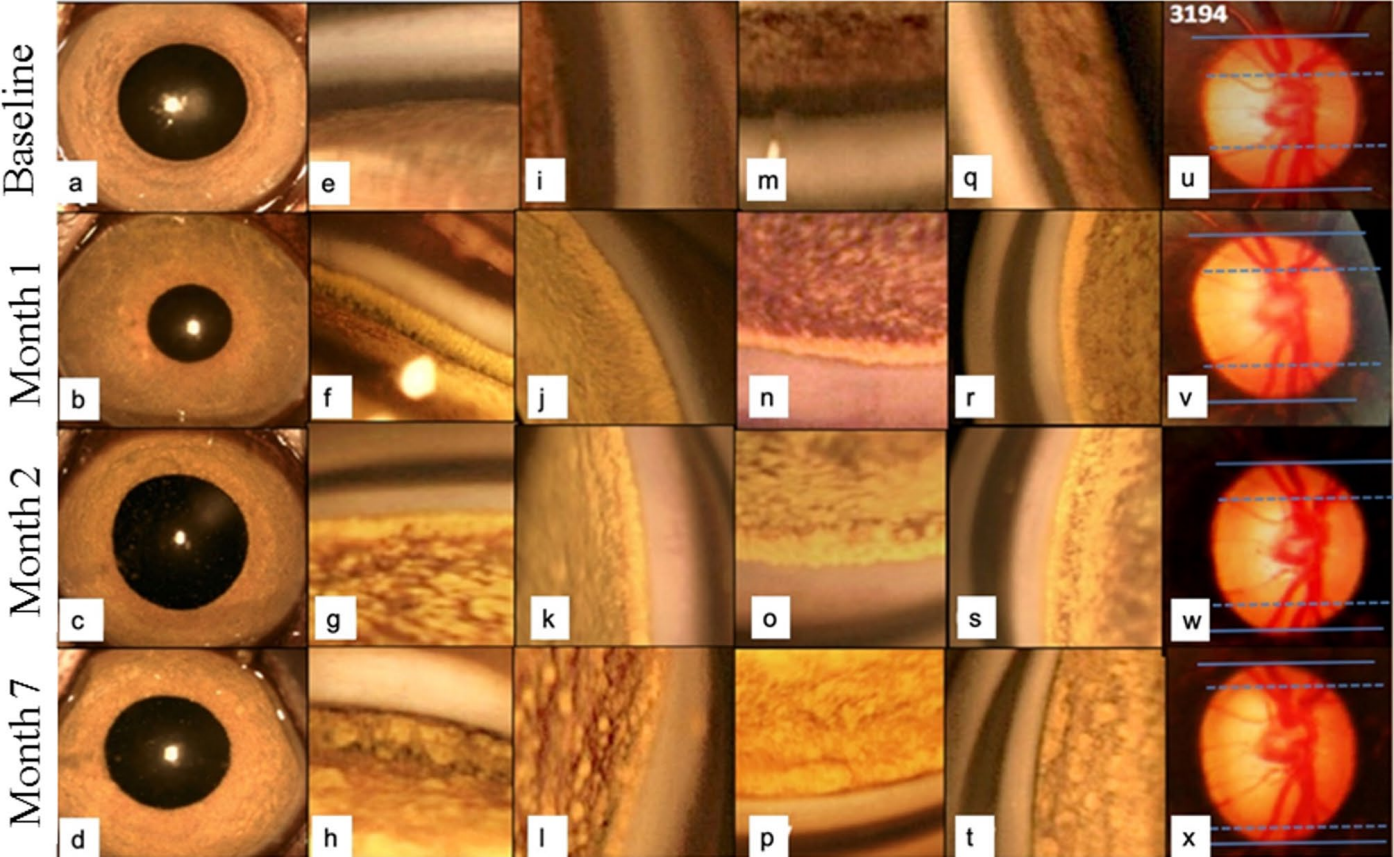
Representative images of primate identification (ID) #3,914. (**a**–**d**) Anterior segment photographs of the right eye of the primate ID 3,914 were included from baseline to month 7. Microbeads were seen on the peripheral iris at month 2 (**c**) and month 7 (**d**). (**e**–**t**) Gonioscopic images showed that the microbeads deposited at the anterior chamber angle of the inferior (**e**–**h**), temporal (**i**–**l**), superior (**m**–**p**) and nasal (**q**–**t**) quadrants of the eye without formation of peripheral anterior synechia at month 7 (**h**,**l**,**p**,**t**). (**u**–**x**) The cupping of the optic nerve head was illustrated with serial fundus photographs.

The microbead injections did not result in persistent cells in the anterior chamber after the first week of injection (Fig. 1). Upon gonioscopic examination, we found that the microbeads occluded the entire 360 degrees of the trabecular meshwork (Fig. 1) after 4 weeks of administering the injections and remained there at month 7. We did not observe any evidence of peripheral anterior synechiae (PAS) or cataract formation at 7 months (Fig. 1). The SD-OCT and disc images were clear and could be monitored from baseline until the end of the study. The corneas of the OHT eyes also remained clear despite the high IOPs (> 40 mmHg) (Table 1).

### Group 1 primates with unilateral IOP elevation

The primates in Group 1 received microbead injections to the right eye and no injections to the contralateral left eye. The mean IOP in the right eye (43 ± 6.3 mmHg) between the period of month 2–7 was significantly higher than its baseline value (12 ± 2.4 mmHg, P < 0.0001, Table 1) as well as the mean IOP of the contralateral control eye (18 ± 2.8 mmHg, P < 0.001, Table 1) between the same period (month 2–7). During months 2–7 (the OHT period), the mean IOP of the left control eyes was higher by an average of 6 mmHg from its baseline IOP (18 ± 2.8 mmHg vs 12 ± 2.2 mmHg, P < 0.001, Table 1) but generally remained < 20 mmHg. The maximum IOP of the OHT right eyes was 54 ± 5.6 mmHg and that of the control left eyes was 20 ± 3.0 mmHg in the Group 1 primates (Table 1).

### Group 2 primates with bilateral IOP elevation

When OHT was induced in both eyes of the Group 2 primates, similar mean IOP elevations were achieved and there was no significant difference between the mean IOP of the right eyes and the left eyes (31 ± 3.2 mmHg vs 31 ± 6.3 mmHg, P = 0.777, Table 1). The mean IOP in both OHT eyes was significantly elevated from their respective baseline values (both P < 0.0001, Table 1). The maximum IOP of the right eyes in Group 2 was significantly lower than that of the Group 1 primates (39 ± 9.2 mmHg vs 54 ± 5.6 mmHg, P = 0.011, Table 1). The maximum IOP was not significantly different between the OHT right and the OHT left eyes in Group 2 (39 ± 9.2 mmHg vs 41 ± 4.7 mmHg, P = 0.702, Table 1). All six primates in Group 2 tolerated the bilateral OHT and remained in good health. There was no loss of body weight or notable change in behaviour in the Group 2 primates when compared to the Group 1 primates.

### Baseline OCT measurements

We found no differences in the LCD, MRW, LC visibility or Bruch’s membrane opening (BMO) area between the two eyes of all primates at baseline (all P > 0.05) (Table 2). The BMO area was consistent in the eyes with OHT from baseline to month 7 (2.64 ± 0.51 mm^2^ vs 2.58 ± 0.48 mm^2^, P = 0.749). The mean visibility of the LC of all eyes at each visit was also consistently between 89 to 92% of the BMO area from enface visualisation (Supplementary Table 1).

**Table 2:** Minimum rim width and Lamina cribrosa depth of each primate at baseline and at 7 months.

ID	Right eye				Left eye			
	Baseline		At 7 months		Baseline		At 7 months	
	MRW, µm (mean ± SD)	LCD, µm (mean ± SD)	MRW, µm (mean ± SD)	LCD, µm (mean ± SD)	MRW, µm (mean ± SD)	LCD, µm (mean ± SD)	MRW, µm (mean ± SD)	LCD, µm (mean ± SD)
**Group 1**								
3914	257.2 ± 85.8	247.5 ± 20.3	111.4 ± 28.3	460.5 ± 44.9	239.8 ± 92.2	248.1 ± 9.71	254.7 ± 50.2	250.3 ± 9.5
5186	317.9 ± 115.4	152.6 ± 7.7	110.3 ± 40.1	207.7 ± 17.9	306.4 ± 108.2	159.8 ± 16.30	299.6 ± 39.8	147.7 ± 18.2
5187	305.0 ± 101.5	202.3 ± 14.5	224.5 ± 48.5	338.7 ± 34.1	312.4 ± 105.7	197.4 ± 13.0	316.7 ± 50.4	195.6 ± 12.1
5189	271.3 ± 107.1	138.0 ± 14.1	257.7 ± 48.0	219.8 ± 20.4	264.2 ± 97.7	162.7 ± 18.3	276.6 ± 48.5	171.3 ± 24.9
5191	192.4 ± 99.1	275.0 ± 6.4	76.1 ± 50.0	584.8 ± 29.6	203.1 ± 91.1	285.8 ± 6.1	208.1 ± 45.9	272.4 ± 12.8
5192	325.2 ± 90.8	188.3 ± 13.3	118.8 ± 24.9	383.7 ± 75.6	312.1 ± 94.6	193.5 ± 14.0	326.8 ± 44.8	183.8 ± 18.3
5194	277.2 ± 111.6	235.3 ± 19.0	227.9 ± 40.6	329.4 ± 34.2	277.23 ± 106.0	227.8 ± 22.2	268.6 ± 52.5	220.9 ± 17.9
5195	276.5 ± 90.9	217.7 ± 10.4	171.3 ± 45.5	284.2 ± 20.0	291.2 ± 92.6	190.5 ± 14.2	298.4 ± 40.9	181.7 ± 21.3
5234	309.2 ± 91.7	158.3 ± 16.4	280.2 ± 40.2	203.7 ± 22.9	278.3 ± 89.5	173.4 ± 21.6	373.95 ± 37.1	164.6 ± 19.1
5235	256.1 ± 92.3	215.5 ± 9.3	92.5 ± 36.1	338.1 ± 16.7	233.4 ± 97.3	215.6 ± 7.6	265.6 ± 52.3	192.3 ± 8.3
Group 1	278.8 ± 39.2	203.0 ± 44.2	167.1 ± 74.9	335.1 ± 120.1	271.8 ± 36.8	205.5 ± 39.8	288.9 ± 45.3	198.1 ± 38.9
**Group 2**								
2677	292.6 ± 43.6	200.7 ± 29.3	242.3 ± 29.9	247.3 ± 43.6	264.3 ± 52.9	192.1 ± 21.3	208.7 ± 48.3	250.2 ± 39.5
2705	293.4 ± 57.3	213.7 ± 33.5	317.3 ± 32.2	252.2 ± 31.7	291.0 ± 53.1	223.6 ± 31.2	270.9 ± 49.7	257.4 ± 32.7
5407	304.7 ± 57.4	236.4 ± 6.7	242.3 ± 17.7	247.3 ± 17.0	289.0 ± 55.3	221.2 ± 14.1	208.7 ± 35.3	250.2 ± 20.4
5408	277.0 ± 47.6	221.4 ± 21.4	317.3 ± 43.8	252.2 ± 24.4	277.5 ± 52.4	225.4 ± 21.2	270.9 ± 52.2	257.35 ± 16.1
2873	341.1 ± 66.6	193.0 ± 33.6	304.2 ± 16.6	262.6 ± 62.6	360.7 ± 62.3	178.1 ± 32.1	182.1 ± 54.3	302.3 ± 46.5
2874	366.2 ± 46.8	197.7 ± 9.2	287.4 ± 42.7	241.6 ± 13.0	372.7 ± 46.4	217.4 ± 4.7	259.2 ± 4.0	248.6 ± 21.4
Group 2	312.5 ± 34.0	210.5 ± 16.5	285.1 ± 34.9	250.5 ± 7.1	309.2 ± 45.7	209.6 ± 19.7	233.4 ± 38.3	261.0 ± 20.6

*ID* primate identification number, *MRW* minimum rim width, *LCD* lamina cribrosa depth, *SD* standard deviation.

### Structural changes of the ONH

In Group 1 primates, the mean MRW of the right eyes with OHT was significantly thinner at month 7 (167.1 ± 74.9 µm) when compared to its baseline value (278.8 ± 39.2 µm, P = 0.00019, Table 2), and that of the control left eyes at the same time point (month 7, 288.9 ± 45.3 µm, P = 0.0110, Table 2). The mean LCD of the right eyes with OHT (335.1 ± 120.1 µm) was also significantly deeper at month 7 when compared to that at baseline (203.0 ± 44.2 µm, P = 0.0011, Table 2), and that of the control left eyes at month 7 (198.1 ± 38.9 µm, P = 0.0049, Table 2).

**Figure 2:**
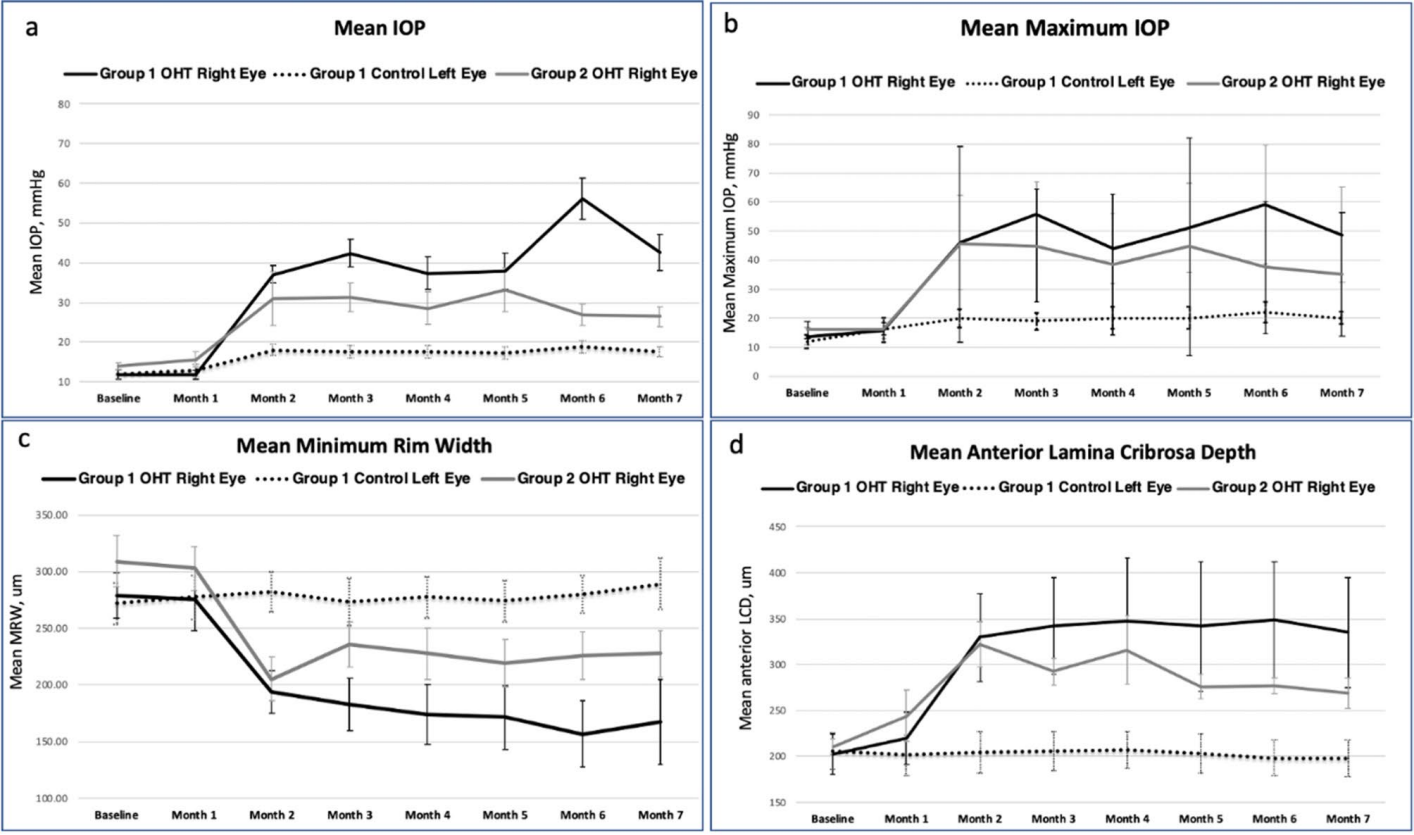
Summary of the intraocular pressure and optical coherence tomography parameters of all primates (N = 16). *OHT* ocular hypertension, *IOP* intraocular pressure, mmHg, *MRW* Minimum Rim Width, *LCD* lamina cribrosa depth. Black lines represent the right OHT eyes of Group 1; the dotted black lines represent the left control eyes of Group 1; Grey lines represent the right OHT eyes of Group 2. (**a**) Mean IOPs of the right eyes of Group 1 and Group 2 were compared with that of the left eyes of Group 1. (**b**) Maximum IOPs of the right eyes of Group 1 and Group 2 were compared with that of the left eyes of Group 1. (**c**) Mean minimum rim width (MRW) of the right eyes of Group 1 and Group 2 were compared with that of the left eyes of Group 1. (**d**) Mean lamina cribrosa depth (LCD) of the right eyes of Group 1 and Group 2 were compared with that of the left eyes of Group 1.

In Group 2 primates, both the right and left OHT eyes showed thinner mean MRW and deeper mean LCD values at month 7 when compared to their baseline values (all P < 0.05, Table 2). The mean changes in MRW and LCD were calculated monthly from baseline to month 7. The greatest changes in the MRW (-93 ± 42 µm) and LCD (86 ± 79.6 µm) values were found in the month 1 to 2 period (both P < 0.001, Table 3) in the right OHT eyes of Groups 1 and 2. Figure 2 compares the MRW, the LCD, mean IOP and maximum IOP values of the right eyes of Group 1 and Group 2 with those of the left eyes of Group 1 as the controls.

**Table 3:** Mean change in minimal rim width and lamina cribrosa depth from baseline to month 7 (end of study).

Monthly time intervals from baseline to month 7	Right eye, OHT, N = 16			Control eye, N = 10			Left eye, OHT, N = 6		
	Mean change in MRW/month	Standard deviation	P value	Mean change in MRW/ month	Standard deviation	P value	Mean change in MRW/month	Standard deviation	P value
Baseline–month 1	− 4.6	31.02	0.5622	5.58	11.4	0.1557	− 8.84	73.6	0.7805
Month 1–month 2	− 93	42	< 0.001	5.14	7.89	0.0697	− 58.93	61.5	0.0656
Month 2–month 3	11.2	39.1	0.2699	− 9.05	16	0.1074	3.99	28	0.7418
Month 3–month 4	− 4.8	18.6	0.3149	3.86	9.79	0.2444	16.4	18.9	0.0873
Month 4–month 5	− 6.3	39.4	0.531	− 3	25	0.713	16.4	18.9	0.0872
Month 5–month 6	− 6.9	41.9	0.5184	5.8	17.6	0.3238	− 2.21	22.5	0.8191
Month 6–month 7	6.58	29.1	0.38	8.78	34.8	0.4453	5.95	10.5	0.2235

Monthly time intervals from baseline to month 7	Mean change in LCD/month	Standard deviation	P value	Mean change in LCD/month	Standard deviation	P value	Mean change in LCD/month	Standard deviation	P value
Baseline–month 1	27.5	44.7	0.0265	− 4.01	8.48	0.1691	33.6	55.7	0.1991
Month 1–month 2	86	79.6	0.0006	3.18	12.3	0.4335	54.2	41.7	0.0245
Month 2–month 3	− 0.5	34.7	0.9525	1.27	4.79	0.4243	− 17.1	58	0.5024
Month 3–month 4	− 1.9	33.8	0.9958	1.28	5.49	0.4804	− 7.49	20.7	0.4155
Month 4–month 5	0.05	40.8	0.5201	− 3.57	19.1	0.5682	− 7.49	20.7	0.4155
Month 5–month 6	− 1	46.7	0.9341	− 5.15	12.2	0.2139	0.75	26.7	0.9481
Month 6–month 7	− 8.4	29.5	0.274	− 0.39	6.32	0.8504	− 16.4	6.05	0.0012

*OHT* ocular hypertension, *MRW* minimum rim width, *LCD* lamina cribrosa depth.

### Correlation of mean and maximum IOP with structural changes of the ONH

**Figure 3:**
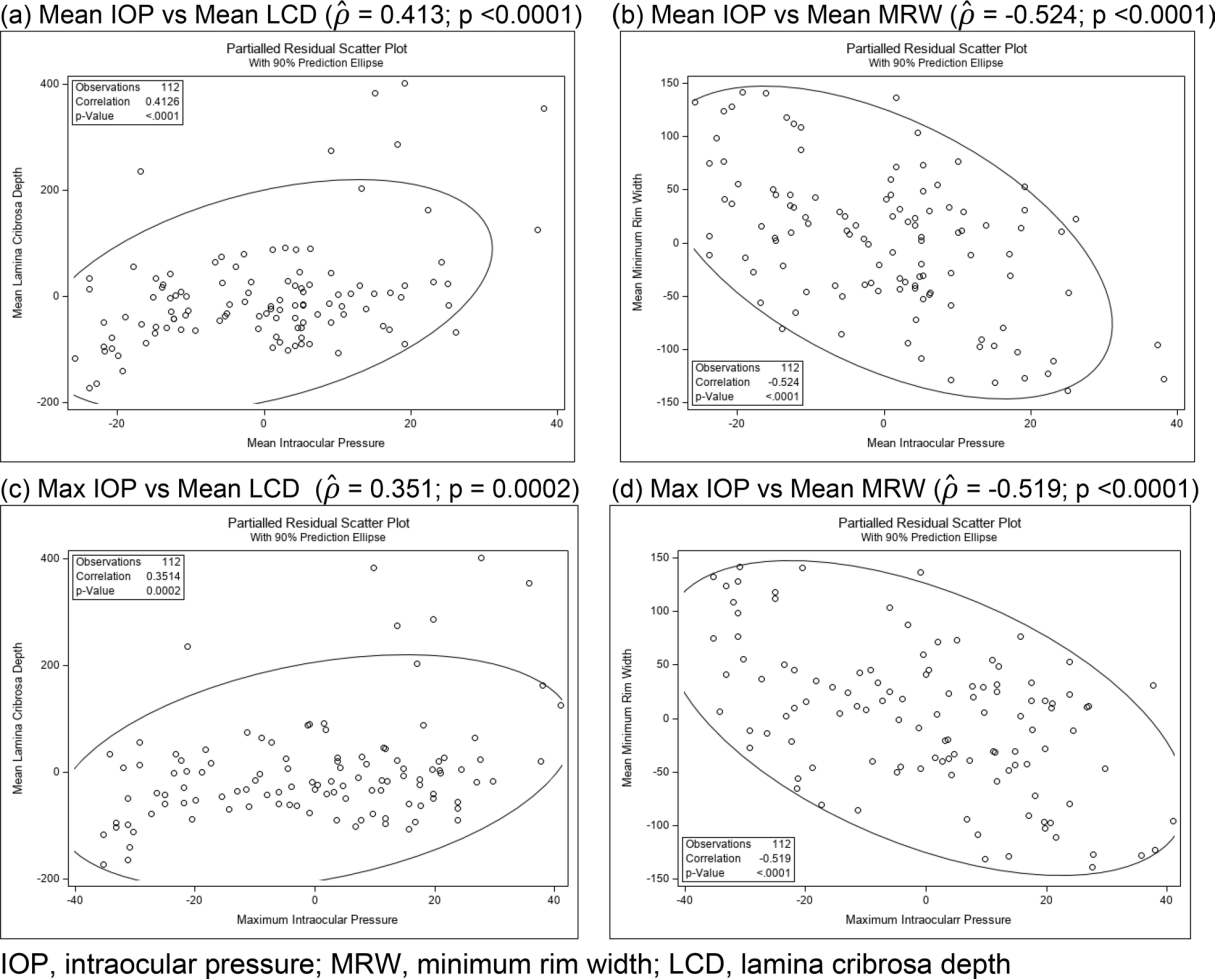
Pearson partial correlation coefficients (${\widehat{\rho }}_{XY|Primate}$) and scatter plots based on the 7 monthly measurements: Mean and maximum IOP (*X*) correlated with the structural changes LCD and MRW (*Y*) of the optic nerve head in the right eyes of Group 1 and 2 primates. (**a**) Mean IOP vs Mean LCD ($\widehat{\rho }$ = 0.413; p < 0.0001). (**b**) Mean IOP vs Mean MRW ($\widehat{\rho }$ = − 0.524; p < 0.0001). (**c**) Max IOP vs Mean LCD ($\widehat{\rho }$ = 0.351; p = 0.0002). (**d**) Max IOP vs Mean MRW ($\widehat{\rho }$ = − 0.519; p < 0.0001). *IOP* intraocular pressure, *MRW* minimum rim width, *LCD* lamina cribrosa depth.

Pearson partial correlation coefficients (${\widehat{\rho }}_{XY|Primate}$), controlling for primate effect, reflecting the level of association between maximum and mean IOP values (*X*) with mean MRW and LCD (*Y*) were calculated using data from the right OHT eye of Group 1 and 2 primates over the 7 monthly measurements. Statistical significance was demonstrated for all four correlations (P < 0.001) (Fig. 3). However, maximum IOP was a slightly better predictor of MRW thinning than mean IOP. Both mean and maximum IOP values correlated slightly better with MRW thinning than LCD deepening (Fig. 3).

## Discussion

In this study, we established the microbead model of OHT in primates and used SD-OCT to assess the early changes that occur at the ONH. We achieved our target IOP in the OHT eyes at 4–5 weeks after 5–6 intracameral injections. We also found that this target IOP could be fine-tuned by modifying the injection protocol. We observed structural changes to the ONH by SD-OCT, including thinning of the MRW and a deepening of the LCD in all eyes with OHT and no change in the control eyes. The greatest structural changes to the ONH occurred between months 1 and 2 in OHT eyes. The mean and maximum IOP values were predictors of these ONH structural changes in OHT eyes. Understanding the ONH changes and associated IOP predictors using OHT models, such as the microbead model proposed herein, could be useful to elucidate glaucoma pathogenesis and to investigate IOP-lowering drugs.

In our hands, we were able to consistently induce OHT in both primate groups using our modified microbead protocol. Although the left eye baseline IOP in the Group 2 was 3 mmHg higher than the Group 1 animals, there was no statistical difference between the Group 2 right and left eye MRW and LCD structural changes. This small IOP difference of 3 mmHg could be multifactorial due to the regional and age difference between our Group 1 animals (imported from Vietnam, older age) and Group 2 animals (which were bred in Singapore, younger age) that may have contributed to minor IOP differences although the primate species was the same. In addition, the measurement of Group 1 and Group 2 IOPs at different time points could have resulted in intra-observer measurement errors^7,18,27^. However, when compared with other primate studies, the baseline IOP in both groups were within reported normal IOP ranges for primates^18,27^.

In the Group 1 primates, we explored the effects of unilateral IOP elevation after following the protocol described by Weber et al*.*^10^ and achieved similar IOP elevations as reported previously. However, the mean and maximum IOP values were higher than the IOP values typically seen in glaucoma patients. To reduce the effects of ischemia that might occur as a result of highly elevated IOP, we modified the protocol by reducing the number of injections and lowering the target IOP to 20 mmHg in our Group 2 primates, with the aim of achieving a lower mean and maximum IOP. With this modified protocol, we were able to reduce the mean IOP in the OHT right eyes from 43 ± 6.3 mmHg (in the Group 1 primates) to 31 ± 3.2 mmHg (in the Group 2 primates). We also reduced the maximum IOP from 54 ± 5.6 mmHg (in the Group 1 primates) to the lower maximum IOP of 39 ± 9.2 mmHg (in the Group 2 primates) (Table 1 and Supplementary Fig. 2). These findings suggest that this approach can induce an elevation in IOP in a more controlled manner than the laser-induction approach, and allows for some degree of titration. Although this model required repeated injections, these were readily performed and did not cause any complications such as inflammation or cataract.

By removing 50 µl of aqueous before injecting the microbeads, we were able to inject a higher volume of beads and reduce the number of injections compared to the Group 1 primates to achieve OHT in the Group 2 primates (Table 1). In the Group 2 primates, we could induce similar intra-animal bilateral IOP elevations (mean IOP right eye, 31 ± 3.2 mmHg and mean IOP left eye, 31 ± 6.3 mmHg, Table 1).

When evaluating the effects and outcomes of a therapy that aims to reduce IOP or confer neuroprotection in OHT eyes, using a different animal as a control could have confounding effects because each animal has a unique susceptibility to IOP^28,29^. Thus, a bilateral primate OHT model might be more useful for assessing the effects of a therapy applied to the study eye, as comparisons can then be made to the contralateral OHT eye which would share a similar susceptibility to the IOP insult^30^. This is the approach that we took in this study with our Group 2 primates. We also previously reported that the bilateral primate OHT model can be established in Association for Assessment and Accreditation of Laboratory Animal Care (AAALAC) International approved laboratories with Institutional Animal Care and Use Committee (IACUC) approval^19^. To maintain the general health and well-being of the primates, strict ethical guidelines were followed in our laboratory. We frequently monitored the body weight, behaviour and food and water intake of the primates and compared these parameters with their counterparts who had not ocular injections. We found no differences in any of these parameters, even in primates with bilateral OHT^19^.

Others have also suggested that the contralateral eye might not be an ideal control for normotension^7,31,32^. Previous data have shown that the contralateral eyes can be affected when the IOP is elevated in the study eyes^33–36^. Some of these changes include microglial activation^37^, transient disc swelling^7^ and longitudinal loss of multifocal electroretinogram signals^32^. In our study, we found that the mean IOP for the contralateral control eyes significantly increased when compared to its mean baseline IOP value in the Group 1 primates, although the mean IOPs in the OHT period between months 2–7 did not exceed 20 mmHg (Table 1). This finding seems to corroborate those of previous studies^33–36^. A consensual IOP rise has been frequently reported in both experimental and clinical glaucoma studies^33–35,37^. Although the exact mechanism for this consensual IOP effect has not been elucidated, postulated mechanisms include: glaucoma drug effects^36^; activation of sympathetic neural signals in the anterior segment that might increase the aqueous outflow^34^; anterior chamber cannulation that activates microglia^37^; and prostaglandin release that might exert a minor effect on the trabecular meshwork and thus cause a minor IOP elevation^35,37^. In our study, we considered that sympathetic neural signals, microglial activation and prostaglandin release might be the most relevant factors that could have caused the consensual IOP rise in our Group 1 primates. Researchers should consider this confounding factor when designing similar studies.

As discussed, we found that the mean changes in MRW (− 93 ± 42 µm) and LCD (86 ± 79.6 µm) were greatest between months 1 and 2 of injection (Fig. 2 and Table 3. It is also within this period that the initial IOP elevation > 20 mmHg or OHT was noted (Table 1). Thus, this particular window might constitute an important period to monitor early ONH structural damage in OHT eyes. Moreover, consistent with previous reports, we also found that the mean and maximum IOP values were significant predictors of MRW and LCD changes in this study (Fig. 3)^24^. Thus, the rise in IOP (between month 1 and 2) might also explain the greatest changes in MRW and LCD during this period (Table 3). Although the greatest changes occurred within the same period in all the primates, the magnitude of the MRW thinning and LCD deepening was less in the Group 2 primates, which had lower mean and maximum IOPs (Fig. 2 and Supplementary Fig. 3). This finding was also reflected in the degree of correlation of these IOP parameters with the magnitude of damage in the ONH. Although both the mean and maximum IOP values were significant predictors of ONH structural changes, the MRW correlation was slightly superior to the LCD in our study (Fig. 3).

We acknowledge several limitations in our study. First, a small sample size (n = 16) was included. However, this is a reasonable sample size for primate studies. Second, our primates were young (4–9 years old). As cynomolgus primates reach sexual maturity between 4 and 6 years of age, some of our older primates could be considered as young adults^38^. Based on the primate-to-human age conversion (ratio 1:3)^39^, our primate ages represent 12–27 human years. Thus, the findings of our study might not be directly applicable to elderly patients with POAG or reflect age-related ONH changes that occur in humans^40^. However, this age group may be a less expensive option to acquire preliminary data because it is increasingly costly and difficult to find older primates. Third, monthly SD-OCT imaging may be too infrequent to capture all of the ONH changes and might only represent a trend change rather than an event-based change^20^. Fourth, we did not re-scale the “x and y” dimensions in the SD-OCT images. The images from the SD-OCT were presumed to be similar to the human eye, and thus rescaling might be needed^4,5,41^. However, the ONH structural changes were not affected by the floor effect. Although axial length may be used to help rescale the SD-OCT image, we did not collect these measurements, which is another limitation of this study. Finally, we did not correlate the ONH structural changes with functional loss determined by electrophysiology approaches, as we had not established the necessary analyses in primates during the study period. We are aware of the importance of performing electrophysiological examinations, as electrophysiological changes precede the manifestation of ONH structural damage; this work will form part of our future research efforts.

In summary, we have characterized the early ONH structural changes in a primate microbead model of OHT. We found that the greatest ONH changes occurred early (within 1–2 months of injection) and that these changes coincided with the first signs of IOP elevation. Importantly, both the mean and maximum IOP values were predictors of ONH structural changes. Although the primate microbead model might require further refinement, we believe that it will be a useful approach for future studies on glaucoma pathogenesis and therapeutic interventions.

## Methods

### Animals

This study was approved and monitored by the SingHealth IACUC. All experiments and animal care procedures were performed in accordance with the Association for Research in Vision and Ophthalmology's (ARVO) Statement for the Use of Animals in Ophthalmic and Vision Research and performed in AAALAC International approved facility. A total of 16 Cynomolgus monkeys (*Macaca fascicularis*) aged 4–9 years old were included in the study (Table 1).

The primates were divided into two groups. Group 1 primates (n = 10) received unilateral IOP elevation, with a target IOP of > 30 mmHg in the right eye whilst the contralateral left eye received no intervention and served as controls. Group 2 primates (n = 6) received bilateral IOP elevation. These primates were induced 12 months after the Group 1 primates, and the aim was to achieve a lower target IOP of > 20 mmHg. For these primates, additional assessments of food and water intake, body weight and behaviour of the primates were performed to ensure that bilateral OHT would not be detrimental to their general well-being.

All procedures were performed under anaesthesia with combinations of ketamine (15 mg/kg) and medetomidine (0.04–0.008 mg/kg) by intramuscular injections. Topical anaesthesia (1–2 drops of 1% Xylocaine) was instilled in both eyes before any contact procedures.

### Chronic IOP elevation with microbead anterior chamber injections

Using a modified version of the protocol by Weber et al*.* for microbead induction of OHT in primates, the IOP was elevated by repeated injection of 15 µm polystyrene microbeads (concentration of 1 million per ml, fluorescent microspheres, Dye-Trak, Triton technology Inc., San Diego, CA) into the anterior chamber. Prior to injection, the beads were spun down and washed in sterile saline and 0.001%Tween 80 to remove any thimerosal preservative. The concentration of the solution doubled to 2 million microbeads per ml. Then, 50 µl of the bead solution was injected slowly over 10–15 min to minimise any IOP spike. The IOP was checked every week (Details in Study parameters). A criterion for re-injection was an IOP < 30 mmHg at any time in Group 1 primates and an IOP < 20 mmHg at any time in Group 2 primates. In Group 2 primates, before the microbead injections, 50–100 µl of aqueous was removed to reduce the risk of IOP spikes. OHT was defined as an IOP > 20 mmHg for more than two consecutive readings.

### Study parameters

The IOP of each eye was measured by TONOVET (Icare, Finland) in the morning (mean of five times per eye) every week after sedating the primates and monthly IOPs were calculated from the weakly measurements. The mean OHT IOP was calculated from the onset of OHT until month 7. Slit-lamp photography (NS-2D, Righton, Japan) and gonioscopic examination were performed to assess the presence of inflammation, cataract and PAS weekly until the IOP elevation was sustained; these assessments were performed monthly thereafter (Fig. 1). Fundus photography (TRC50Dx, Topcon, Japan) and SD-OCT imaging (Spectralis, Heidelberg Engineering, Germany) were performed monthly. Each OCT volume consisted of 73 serial horizontal B-scans (62 µm distance between B-scans; 384 A-scans per B-scan) that covered a square area of 15° × 15° centred on the ONH. The eye-tracking and enhanced depth imaging (EDI) modalities of the Spectralis software were used during image acquisition. Each B-scan was averaged 50 times during acquisition (Supplementary Fig. 1)^6,22,39^.

### OCT image enhancement, delineation, 3D reconstruction and analyses

Raw SD-OCT images were enhanced using an adaptive compensation algorithm to remove blood vessel shadows, enhance tissue contrast, improve the visibility of the LC, and reduce noise over-amplification in the deepest layers of the ONH^4,5,39^. Segmentation of the post-processed SD-OCT volume of each eye was performed using our previously reported custom-written MATLAB (MathWorks Inc., Natick, MA) algorithms^6,22^. The anterior LC and BMO were manually marked while the internal limiting membrane (ILM) was delineated automatically from the Spectralis software. The anterior LC surface was defined by a sharp increase in axial signal intensity (corresponding to collagen) extending laterally up to the LC insertion points in the peripapillary sclera^4,6^. The BMO was defined as the endpoint of the Bruch's membrane layer on either side of the ONH (Supplementary Fig. 1)^4^. The ONH structures were then reconstructed in 3D, and the LCD was defined as the perpendicular distance from the BMO reference plane to the anterior LC surface^22^. All LCD values of the reconstructed 3D ONH were averaged and reported as the mean LCD (Supplementary Fig. 1). The MRW was defined as the shortest distance from the BMO to the ILM. A constraint was applied to ensure that the MRW measurements remained within the neuroretinal rim (bounded by BMO). The mean MRW was reported and compared between eyes with OHT and controls (Fig. 2). The visibility of the LC was calculated as the percentage of the BMO area from the enface visualization^6,22^.

### Statistical analyses

Statistical analyses were performed using SAS (Version 9.3, SAS Institute Inc., Cary, NC, USA). A repeated measures mixed-effects model analysis (SAS PROC GLIMMIX) was used to compare the IOP, LCD and MRW of the eyes with OHT and controls at each visit. The mean and maximum IOP value was calculated from the weekly IOP readings and averaged over each monthly intervals from baseline to months 7 (end-of-study time point). The mean MRW and LCD values were calculated in the same monthly interval. Longitudinal changes in MRW and LCD over 7 monthly measurements were correlated with monthly mean and maximum IOP values using the Pearson partial correlation coefficient, controlling for primate effect, to determine which IOP parameters were predictive of ONH structural changes. Fisher's z-transform of the partial correlation was used to test the null hypothesis of partial correlation equal to zero versus the alternative of not equal to zero. Statistical significance was set at P < 0.05.

## Supplementary information

Supplementary Information.

## References

[1] Kapetanakis VV (2016). Global variations and time trends in the prevalence of primary open angle glaucoma (POAG): a systematic review and meta-analysis. Br. J. Ophthalmol..

[2] Jonas JB (2017). Glaucoma. Lancet.

[3] Fortune B (2016). Experimental glaucoma causes optic nerve head neural rim tissue compression: a potentially important mechanism of axon injury. Invest. Ophthalmol. Vis. Sci..

[4] Strouthidis NG, Fortune B, Yang H, Sigal IA, Burgoyne CF (2011). Longitudinal change detected by spectral domain optical coherence tomography in the optic nerve head and peripapillary retina in experimental glaucoma. Invest. Ophthalmol. Vis. Sci..

[5] Strouthidis NG (2010). A comparison of optic nerve head morphology viewed by spectral domain optical coherence tomography and by serial histology. Invest. Ophthalmol. Vis. Sci..

[6] Tun TA (2016). Shape changes of the anterior lamina cribrosa in normal, ocular hypertensive, and glaucomatous eyes following acute intraocular pressure elevation. Invest. Ophthalmol. Vis. Sci..

[7] Burgoyne CF (2015). The non-human primate experimental glaucoma model. Exp. Eye Res..

[8] Calkins DJ, Lambert WS, Formichella CR, McLaughlin WM, Sappington RM (2018). The microbead occlusion model of ocular hypertension in mice. Methods Mol. Biol..

[9] Rasmussen CA, Kaufman PL (2005). Primate glaucoma models. J Glaucoma.

[10] Weber AJ, Zelenak D (2001). Experimental glaucoma in the primate induced by latex microspheres. J. Neurosci. Methods.

[11] Raghunathan V (2017). Biomechanical, ultrastructural, and electrophysiological characterization of the non-human primate experimental glaucoma model. Sci. Rep..

[12] Samsel PA, Kisiswa L, Erichsen JT, Cross SD, Morgan JE (2011). A novel method for the induction of experimental glaucoma using magnetic microspheres. Invest. Ophthalmol. Vis. Sci..

[13] Sappington RM, Carlson BJ, Crish SD, Calkins DJ (2010). The microbead occlusion model: a paradigm for induced ocular hypertension in rats and mice. Invest. Ophthalmol. Vis. Sci..

[14] Urcola JH, Hernandez M, Vecino E (2006). Three experimental glaucoma models in rats: comparison of the effects of intraocular pressure elevation on retinal ganglion cell size and death. Exp. Eye Res..

[15] Lambert WS (2019). Towards a microbead occlusion model of glaucoma for a non-human primate. Sci. Rep..

[16] Iwasaki K, Uno Y (2009). Cynomolgus monkey CYPs: a comparison with human CYPs. Xenobiotica.

[17] McDonald TF, Cheeks L, Slagle T, Green K (1991). Marijuana-derived material-induced changes in monkey ciliary processes differ from those in rabbit ciliary processes. Curr. Eye Res..

[18] Bito LZ, Merritt SQ, DeRousseau CJ (1979). Intraocular pressure of rhesus monkey (*Macaca mulatta*). I. An initial survey of two free-breeding colonies. Invest. Ophthalmol. Vis. Sci..

[19] Chan ASY, Tun SBB, Barathi VA, Aung T (2018). Bilateral intraocular pressure (IOP) changes in a non human primate (NHP) microbead model of chronic IOP elevation: can both eyes achieve similar elevations for therapeutics evalution?. Invest. Ophthalmol. Vis. Sci..

[20] He L (2014). Longitudinal detection of optic nerve head changes by spectral domain optical coherence tomography in early experimental glaucoma. Invest. Ophthalmol. Vis. Sci..

[21] Yang H (2011). Posterior (outward) migration of the lamina cribrosa and early cupping in monkey experimental glaucoma. Invest. Ophthalmol. Vis. Sci..

[22] Tun TA (2015). Determinants of optical coherence tomography-derived minimum neuroretinal rim width in a normal Chinese population. Invest. Ophthalmol. Vis. Sci..

[23] Gardiner SK (2015). Structural measurements for monitoring change in glaucoma: comparing retinal nerve fiber layer thickness with minimum rim width and area. Invest. Ophthalmol. Vis. Sci..

[24] Gardiner SK, Fortune B, Wang L, Downs JC, Burgoyne CF (2012). Intraocular pressure magnitude and variability as predictors of rates of structural change in non-human primate experimental glaucoma. Exp. Eye Res..

[25] Nouri-Mahdavi K (2004). Predictive factors for glaucomatous visual field progression in the Advanced Glaucoma Intervention Study. Ophthalmology.

[26] Quigley HA, Guy J, Anderson DR (1979). Blockade of rapid axonal transport. Effect of intraocular pressure elevation in primate optic nerve. Arch. Ophthalmol..

[27] Fortune B, Burgoyne CF, Cull G, Reynaud J, Wang L (2013). Onset and progression of peripapillary retinal nerve fiber layer (RNFL) retardance changes occur earlier than RNFL thickness changes in experimental glaucoma. Invest. Ophthalmol. Vis. Sci..

[28] Burgoyne CF, Downs JC, Bellezza AJ, Suh JK, Hart RT (2005). The optic nerve head as a biomechanical structure: a new paradigm for understanding the role of IOP-related stress and strain in the pathophysiology of glaucomatous optic nerve head damage. Prog. Retin Eye Res..

[29] Yang H (2011). Deformation of the early glaucomatous monkey optic nerve head connective tissue after acute IOP elevation in 3-D histomorphometric reconstructions. Invest. Ophthalmol. Vis. Sci..

[30] Jampel HD, Thibault D, Leong KW, Uppal P, Quigley HA (1993). Glaucoma filtration surgery in nonhuman primates using taxol and etoposide in polyanhydride carriers. Invest. Ophthalmol. Vis. Sci..

[31] Fortune B, Cull G, Reynaud J, Wang L, Burgoyne CF (2015). Relating retinal ganglion cell function and retinal nerve fiber layer (RNFL) retardance to progressive loss of RNFL Thickness and optic nerve axons in experimental glaucoma. Invest. Ophthalmol. Vis. Sci..

[32] Fortune B, Cull G, Wang L, Van Buskirk EM, Cioffi GA (2002). Factors affecting the use of multifocal electroretinography to monitor function in a primate model of glaucoma. Doc. Ophthalmol..

[33] Diestelhorst M, Krieglstein G (1991). The effect of trabeculectomy on the aqueous humor flow of the unoperated fellow eye. Graefes Arch. Clin. Exp. Ophthalmol..

[34] Gibbens MV (1988). Sympathetic influences on the consensual ophthalmotonic reaction. Br. J. Ophthalmol..

[35] Kaushik S (2016). Change in intraocular pressure in the fellow eye after glaucoma surgery in 1 eye. J. Glaucoma.

[36] Piltz J (2000). Contralateral effect of topical beta-adrenergic antagonists in initial one-eyed trials in the ocular hypertension treatment study. Am. J. Ophthalmol..

[37] Kezic JM, Chrysostomou V, Trounce IA, McMenamin PG, Crowston JG (2013). Effect of anterior chamber cannulation and acute IOP elevation on retinal macrophages in the adult mouse. Invest. Ophthalmol. Vis. Sci..

[38] Xie L (2013). Age- and sex-based hematological and biochemical parameters for *Macaca fascicularis*. PLoS ONE.

[39] Kohama SG, Rosene DL, Sherman LS (2012). Age-related changes in human and non-human primate white matter: from myelination disturbances to cognitive decline. Age.

[40] Yang H (2014). Age-related differences in longitudinal structural change by spectral-domain optical coherence tomography in early experimental glaucoma. Invest. Ophthalmol. Vis. Sci..

[41] Strouthidis NG, Fortune B, Yang H, Sigal IA, Burgoyne CF (2011). Effect of acute intraocular pressure elevation on the monkey optic nerve head as detected by spectral domain optical coherence tomography. Invest. Ophthalmol. Vis. Sci..

