# Supplementary material for: Longitudinal assessment of optic nerve head changes using optical coherence tomography in a primate microbead model of ocular hypertension
Source: Sci Rep. 2020 Sep 7;10:14709. doi: 10.1038/s41598-020-71555-0 (PMC7477239; doi:10.1038/s41598-020-71555-0)
Supplement: Supplementary file 1 — Supplementary Information. [file 41598_2020_71555_MOESM1_ESM.docx]

**Supplementary Figure 1:** Measurements of depth of anterior lamina cribrosa and Bruch’s membrane opening-minimum rim width on a reconstructed 3-dimensional optic nerve head

**
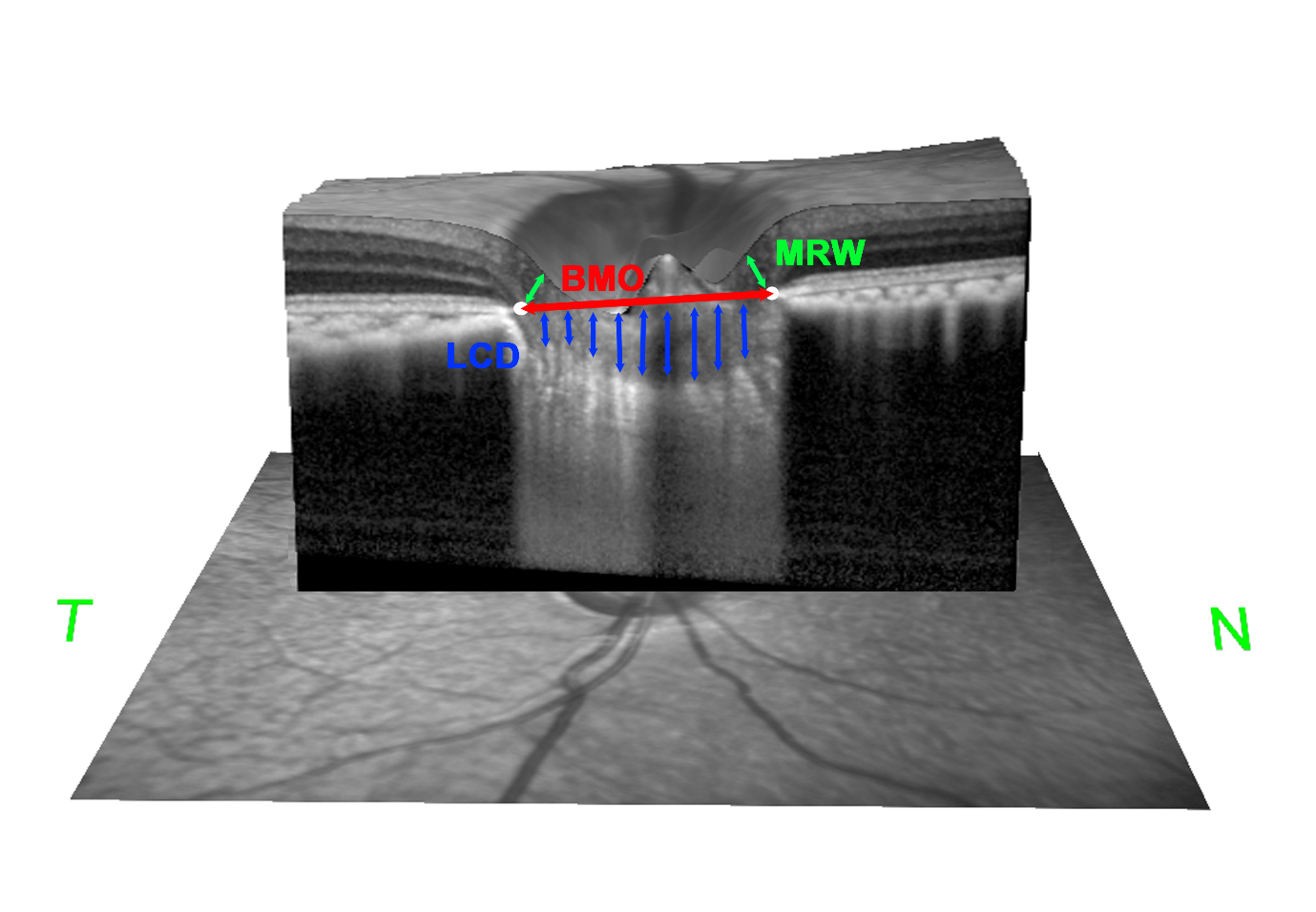
**

Blue arrows show the lamina cribrosa depth (LCD) from the Bruch’s membrane opening (BMO, the reference plane, red line) and green lines show the minimum rim width (MRW).

**Supplementary Figure 2: Intraocular pressure profiles of each primate**


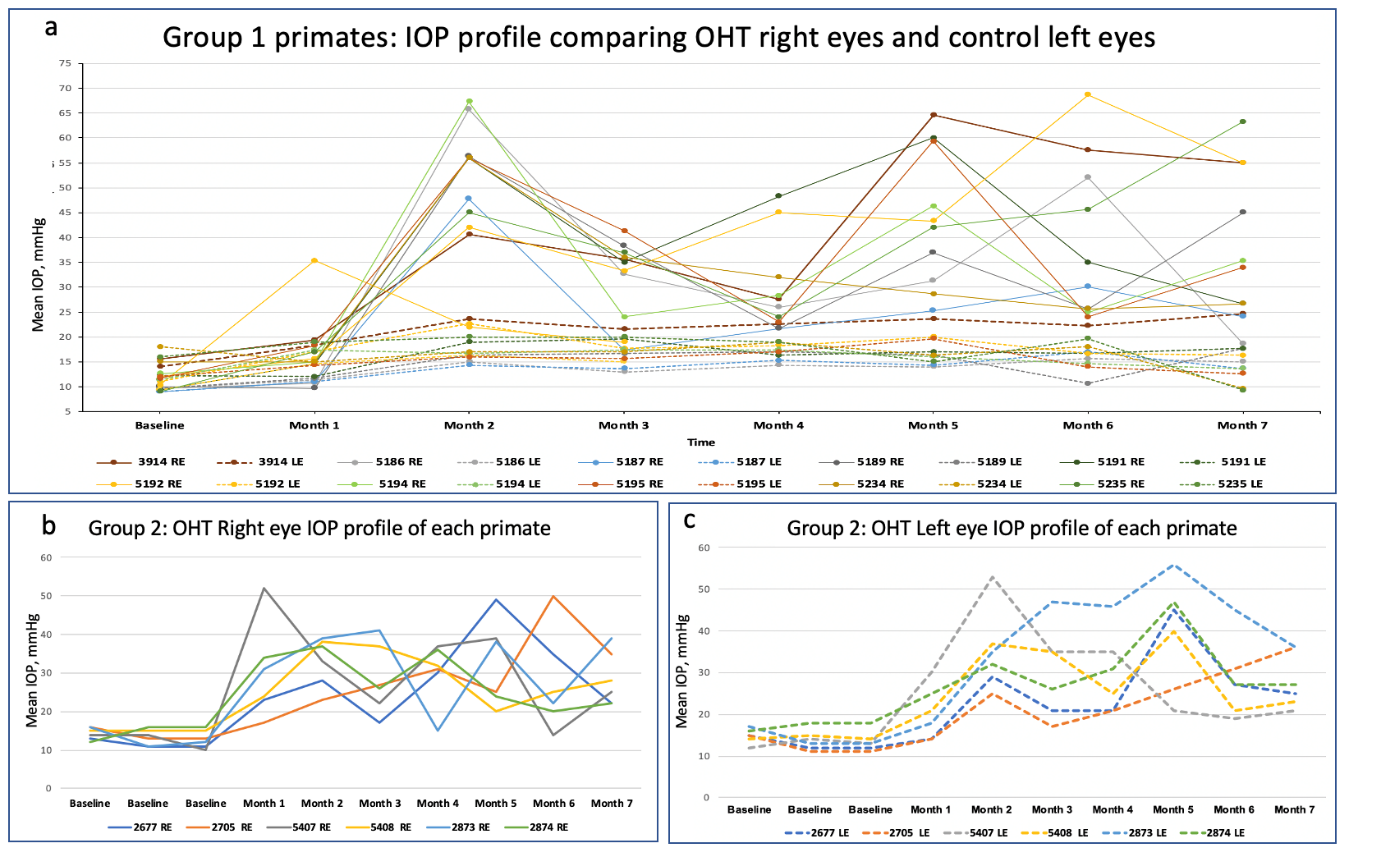


IOP, Intraocular pressure, mmHg; OHT, ocular hypertension; RE, right eye; LE, left eye.

**Supplementary Figure 3: Longitudinal Lamina cribrosa depth and minimum rim width changes of each ocular hypertension primate and their controls**

**
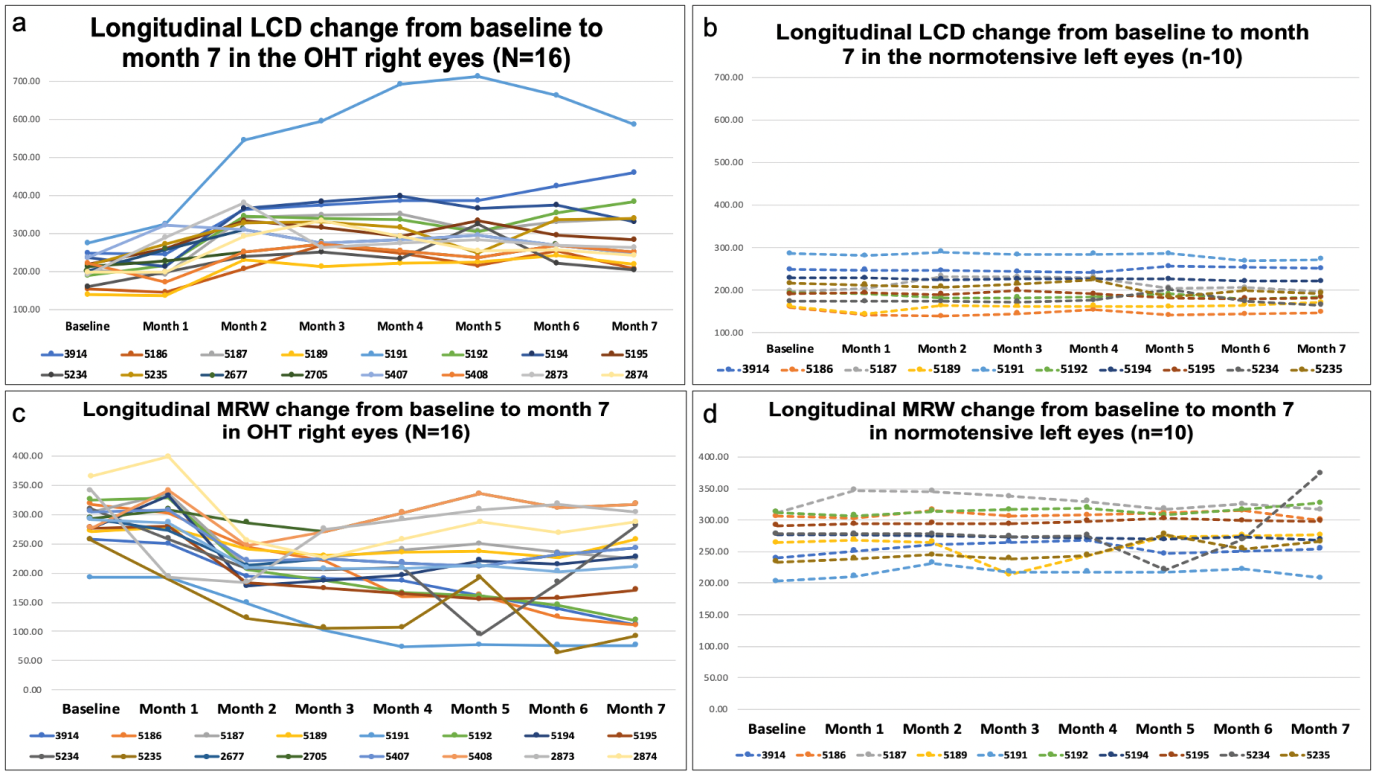
**

OHT, ocular hypertension; MRW, minimum rim width; LCD, lamina cribrosa depth.

**Supplementary Table 1:** Baseline imaging characteristics of the primates (N=16)

| **Variables** | **Right Eyes** | **Left Eyes** | **P value** |
| --- | --- | --- | --- |
| **Mean LCD, µm** | 205.8 ± 35.7 | 207.0± 32.9 | 0.922 |
| **Mean MRW, µm** | 291.4 ± 39.9 | 285.8 ±43.1 | 0.705 |
| **Mean BMO area, mm^2^** | 2.64 ± 0.51 | 2.61± 0.49 | 0.877 |
| **LC visibility, %** | 92.2 ± 4.10 | 91.3± 4.76 | 0.550 |

LCD is lamina cribrosa depth; BMO is Bruch’s membrane opening; MRW is minimum rim width; the data are shown as mean ± standard deviation; LC visibility was calculated as the visibility of lamina cribrosa (LC) as the percentage of BMO area from enface visualization.
